# Elevated Ki-67 (MIB-1) expression as an independent predictor for unfavorable pathologic outcomes and biochemical recurrence after radical prostatectomy in patients with localized prostate cancer: A propensity score matched study

**DOI:** 10.1371/journal.pone.0224671

**Published:** 2019-11-07

**Authors:** Seok-Soo Byun, Minseung Lee, Sung Kyu Hong, Hakmin Lee

**Affiliations:** Department of Urology, Seoul National University Bundang Hospital, Seongnam, Korea; The Cancer Institute of New Jersey, Robert Wood Johnson Medical School, UNITED STATES

## Abstract

**Background:**

Ki-67 is known to be useful in estimating the fraction of proliferation tumor cells in various malignancies. We tried to investigate clinical association of Ki-67 (MIB-1) expression with the oncological outcomes in patients with localized prostate cancer (PCa) after the radical prostatectomy (RP).

**Materials and Methods:**

We retrospectively analyzed the data of 1,561 patients who underwent RP for localized PCa. According to the propensity score having Ki-67 expression, 183 patients with positive Ki-67 expression were matched to 549 patients without Ki-67 expression. By using multivariate Cox-proportional hazards models and logistic regression tests, the prognostic value of each variable was tested.

**Results:**

After propensity score matching, positive Ki-67 group showed significant worse clinical characteristics and pathologic outcomes than negative Ki-67 group. The multivariate analysis showed that the Ki-67 expression was significantly associated with several adverse pathologic outcomes including higher pathologic stage (p = 0.006), higher grade group (p = 0.005), seminal vesicle invasion (p = 0.036), and positive surgical margin (p = 0.025). The group with Ki-67 expression showed significant worse biochemical recurrence-free survival (p<0.001) than negative Ki-67 group. Subsequent multivariate Cox analyses showed that Ki-67 was independent predictor for BCR after RP (HR 1.549, 95% CI 1.187–2.021, p = 0.001).

**Conclusion:**

In our study, high Ki-67 expression was significantly related with adverse pathological and finally with worse biochemical recurrence-free survival. Further studies are needed to validate the prognostic value of Ki-67 more exactly in PCa patients.

## Introduction

Prostate cancer is the most frequently diagnosed malignancy in United States, and the fifth common malignancy in the world [1]. As the radical treatment such as radical prostatectomy or radiation therapy lead to significant functional impairments such as urinary incontinence and erectile dysfunction, conservative treatment such as active surveillance or watchful waiting gains more strength as a reliable substitute [2]. Therefore, careful and appropriate selection of treatment is more important than ever. Reliable biomarker which can predict the prognosis of disease is valuable for aforementioned reasons. The recent advance in molecular biology also facilitates the development of commercial genetic biomarkers but a reliable biomarker is still lacking [3]. The Ki-67 protein is well known and widely utilized as a proliferation marker of tumor cells [4]. Although the function of Ki-67 antigen is still not unknown, but Ki-67 antigens are exclusively present only in proliferating cells. The proportion of Ki-67 protein in tumor cells expected to have a prognostic value in several different types of malignancy. Several studies reported Ki-67 as a prognostic factor of survival and biochemical recurrence of prostate cancer, but most of their study limited by small number and heterogeneity of subjects [5–8]. In the present study, we aimed to evaluate the prognostic impact of Ki-67 expression after radical prostatectomy in patients with clinically localized prostate cancer using the propensity score matching method.

## Materials and methods

The study protocol was approved by the institutional review board of Seoul National University Bundang Hospital (IRB No. B-1801-445-102). Informed consent was waived by the institutional review board. We analyzed the data of 1,727 patients who underwent RP from May 2006 to December 2014 at our institution. After additional exclusion of patients (preoperative hormone therapy [n = 21], other malignancies [n = 110] and incomplete information [n = 35]), we finally analyzed a total of 1,561 patients. Patients’ clinical and pathological information were retrieved from our institutional database which is prospectively maintained. All the surgical specimens were reviewed and classified by our uro-pathologist who was unaware of any clinical information about patients by using the modified definition of the 2005 international society of urological pathology consensus conference [9]. The TNM staging was evaluated by using the 6^th^ edition of the American Joint Committee cancer guidelines [10]. The pathologic information including immunohistochemistry was also collected prospectively, and analyzed retrospectively. The biochemical recurrence (BCR) was defined as an elevation of prostate specific antigen over 0.2 ng/dl in two sequential examinations. The postoperative follow-ups were usually performed at 3- to 6-month intervals at the initial 2 years and yearly thereafter.

### Immunohistochemical assay

Specimens were fixed in 20% buffered formalin, processed and embedded in paraffin. After sectioned by 5-mm interval, specimens were de-paraffinized in xylene and re-hydrated in graded ethanol. Hematoxylin and eosin staining and Ki-67 staining was subsequently performed. Ki-67 immunohistochemistry staining was performed by streptavidin-biotin technique using the Ki-67 monoclonal antibody (MIB-1, DAKO corporation, Carpinteria, CA). The de-paraffinization of specimen sections was placed into Perixidase Blocking Solution (DAKO, CA, USA) for 5 minutes, and incubation was performed with primary antibody for one hour. After incubation, sections were counterstained by hemallume. The whole sections were inspected and the expression of Ki-67 was determined under light microscopic inspection by single pathologist. If there were multiple tumors with different expression level in Ki-67, we regarded the highest value as the representatives. When we analyzed the ROC curves of Ki-67 on BCR, the cut-off of 10% was revealed to have the best discriminating ability. Therefore, the patients were considered to have positive expression of Ki-67 when more than 10% of the tumor cells were positively stained by Ki-67 antigen.

### Statistical analyses

As there were significant differences in several preoperative characteristics, we performed propensity score matching analysis based on the propensity to have positive Ki-67 expression. The propensity scores were calculated using non-parsimonious multi-variate logistic regression tests according to preoperative characteristics including age, body mass index (BMI), type of surgery, history of hypertension, history of diabetes mellitus, prostatic specific antigen (PSA) level, clinical stage, and biopsy grade group. After excluding 32 patients without appropriate pairs, 183 patients with positive Ki-67 expression were matched to 549 patients without Ki-67 expression by a 1:3 ratio using the nearest neighbour method with a calibre of 0.02. Independent t-tests and chi-square tests were performed to compare the clinical or pathological characteristics between the groups. Uni–and multivariates logistic regression tests and Cox proportional hazard models were utilized to evaluate possible clinical associations on pathological and clinical outcomes. Kaplan–Meier tests with log-rank analysis were performed for evaluating the survival outcomes between the two subgroups by Ki-67. All of the statistical analyses were performed by SPSS software (SPSS 19.0, Chicago, IL, USA). All the p-values presented as two-sided value and p < 0.05 was considered statistically significant.

## Results

The characteristics of entire patients were summarized in the Table 1. Median age at surgery were 67.0 [Interquartile range (IQR) 62.0–71.0] years, and median PSA were 7.7 (IQR 5.1–13.5) ng/ml. Median duration of follow up was 56.0 months (IQR 28.0 to 83.0). There were 215 (13.8%) patients with positive Ki-67 expression and 1,337 (85.7%) patients without Ki67 expression. Positive Ki-67 group showed significantly higher prostate specific antigen (PSA) level (p<0.001), worse clinical stage (p < 0.001), and higher biopsy grade group (p < 0.001) than negative group. The positive Ki-67 group was revealed to have significantly worse pathologic outcomes in terms of pathologic stage, pathologic grade group, rate of extracapsular extension (ECE), rate of seminal vesicle invasion (SVI), and positive surgical margin (PSM) (p < 0.001).

**Table 1:** Clinical and pathologic characteristics by Ki-67 expression.

Parameters	Before propensity score matching (mean value or counts (percent))				After propensity score matching (mean value or counts (percent))			
	Ki-67 positive (n = 215)	Ki-67 negative (n = 1,346)	SMD	p value	Ki-67 positive (n = 183)	Ki-67 negative (n = 549)	SMD	p value
Median Age (y)	66.3	65.6	0.105	0.169	66.0	66.2	0.043	0.619
Median BMI (*kg*/*m*^2^)	24.3	24.7	0.054	0.562	24.4	24.1	0.119	0.157
Laparoscopy (yes)	65 (30.3%)	775 (57.3%)	0.662	< 0.001	61 (33.3%)	175 (32.1%)	0.027	0.953
Diabetes mellitus	34 (15.8%)	203 (15.1%)	0.020	0.760	31 (16.9%)	86 (15.7%)	0.035	0.771
PSA	17.9	11.6	0.328	< 0.001	13.8	9.0	0.049	0.586
Prostate volume	38.5	38.0	0.033	0.655	38.2	39.1	0.056	0.524
Clinical stages			0.609	< 0.001			0.027	0.953
cT1	69 (32.1%)	819 (60.8%)			67 (36.6%)	195 (35.5%)		
cT2	146 (68.0%)	527 (39.2%)			110 (61.1%)	337 (61.4%)		
≥ cT3					6 (3.3%)	17 (3.1%)		
Biopsy grade group			0.715	< 0.001			0.11	0.438
1	53 (24.7%)	670 (49.8%)			53 (29.0%)	185 (33.7%)		
2–3	79 (36.7%)	516 (38.3%)			79 (43.2%)	230 (41.9%)		
≥ 4	83 (38.6%)	160 (11.9%)			51 (27.9%)	134 (24.4%)		
Pathologic grade group				< 0.001				0.003
1	13 (6.0%)	249 (18.5%)			13 (7.1%)	86 (15.7%)		
2–3	132 (61.4%)	974 (72.4%)			125 (68.3%)	371 (65.6%)		
≥ 4	70 (32.6%)	123 (9.1%)			45 (24.6%)	92 (16.8%)		
Pathologic stages				< 0.001				0.001
pT2	91 (42.3%)	950 (70.6%)			86 (47.0%)	337 (61.4%)		
pT3	115 (53.5%)	378 (28.1%)			92 (50.3%)	197 (35.9%)		
pT4	9 (4.2%)	18 (1.3%)			5 (2.7%)	15 (2.7%)		
ECE	120 (55.8%)	389 (28.9%)		< 0.001	93 (50.8%)	209 (38.1%)		0.003
SVI	53 (7.4%)	99 (24.7%)		< 0.001	37 (20.2%)	60 (10.9%)		0.002
PSM	110 (51.2%)	380 (28.2%)		< 0.001	86 (47.0%)	183 (33.3%)		0.001

PSA, prostate specific antigen; BMI, Body mass index; ECE, extracapsular extension; SVI, seminal vesicle invasion; PSM, positive surgical margin; SMD, Standardized mean differences

### Before propensity score matching.

**Fig 1:**
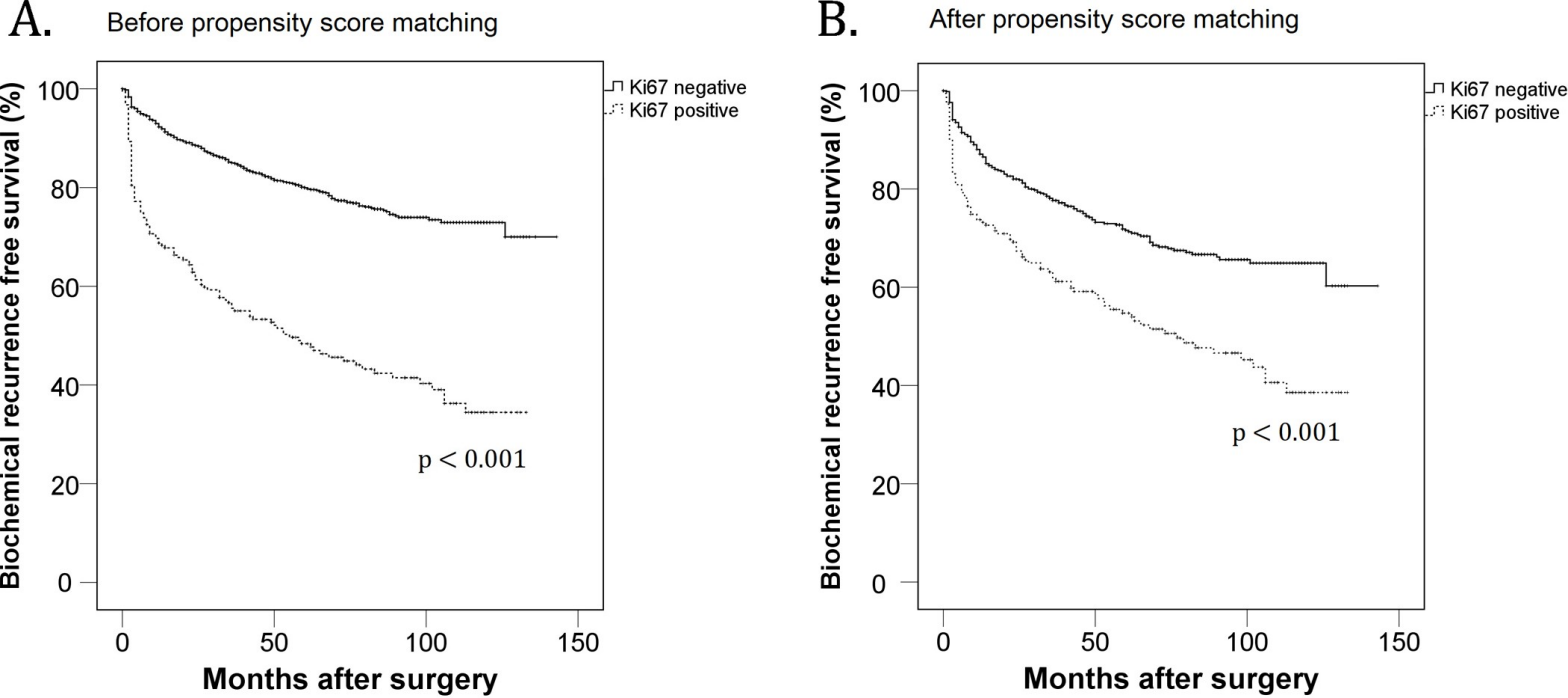
Kaplan-Meier analysis for biochemical recurrence free survival according to the expression of Ki-67 among the entire 1,561 patients (A) and among the 732 patients after propensity scored matching (B).

From the multivariate regression tests, the expression of Ki-67 was significantly associated with having following adverse pathologic outcomes [pathologic grade group (≥ 3) (HR 3.302, 95% CI 2.397–4.548, p < 0.001), pathologic stage (≥T3) (HR 2.849, 95% CI 2.065–3.929, p < 0.001), ECE (HR 2.849, 95% CI 1.944–3.699, p < 0.001), SVI (HR 3.491, 95% CI 2.317–5.260, p < 0.001), and PSM (HR 2.343, 95% CI 1.713–3.205, p < 0.001)(Table 2)]. The multivariate regression tests were adjusted by following co-variables: age, prostate specific antigen, body mass index, prostate volume, history of diabetes mellitus and hypertension, pathologic stage and pathologic grade group. The Kaplan-Meier analyses revealed that the positive Ki-67 group had significantly inferior BCR-free survival than the negative Ki-67 group (p < 0.001) (Fig 1). Subsequent multivariate Cox proportional analyses revealed that the Ki-67 expression is an independent predictor for worse BCR-free survival after surgery (HR 1.577, 95% CI 1.249–1.992, p < 0.001) (Table 3). When we analyzed the Ki-67 expression as a continuous variable, the Ki-67 expression was also revealed as independent predictor for BCR from Cox-proportional analyses (HR 1.024, 95% CI 1.010–1.037, p = 0.001) (S1 Table).

**Table 2:** Multivariate analyses for the impact of Ki-67 on various pathologic outcomes after surgery.

	Before propensity score matching			After propensity score matching		
	HR	95% CI of HR	p-value	HR	95% CI of HR	p-value
Pathologic grade group (≥3)	3.302	2.397–4.548	< 0.001	1.718	1.177–2.507	0.005
Pathologic stage (≥T3)	2.849	2.065–3.929	< 0.001	1.707	1.162–2.507	0.006
Extracapsular extension	2.682	1.944–3.699	< 0.001	0.393	0.089–1.741	0.218
Seminal vesicle invasion	3.491	2.317–5.260	< 0.001	1.826	1.041–3.202	0.036
Positive surgical margin	2.343	1.713–3.205	< 0.001	1.568	1.057–2.325	0.025

Multivariate analyses were adjusted for age, prostatic specific antigen, body mass index, prostate volume, history of diabetes mellitus, history of hypertension, pathologic grade group and pathologic stage. HR = hazard ratio; CI = confidence interval.

**Table 3:** Multivariate analyses using Cox proportional hazard model on biochemical recurrence after propensity score matching (Ki-67 as categorical variable).

	Before propensity score matching			After propensity score matching		
	HR	95% CI	p value	HR	95% CI	p value
Age	0.997	0.982–1.013	0.737	1.007	0.986–1.028	0.518
BMI	1.004	0.996–1.013	0.313	1.007	0.957–1.060	0.782
Diabetes mellitus	1.165	0.894–1.519	0.258	1.175	0.852–1.619	0.325
Hypertension	0.928	0.756–1.140	0.478	0.951	0.731–1.239	0.711
Log (PSA)	1.496	1.315–1.702	< 0.001	1.632	1.371–1.942	< 0.001
Prostate volume	0.994	0.986–1.101	0.102	0.989	0.979–0.999	0.032
High Ki-67	1.577	1.249–1.992	< 0.001	1.549	1.187–2.021	0.001
Pathologic grade group						
Group 1	Reference			Reference		
Group 2–3	2.924	1.610–5.310	< 0.001	2.447	1.118–5.356	0.025
Group ≥ 4	6.894	3.640–13.056	< 0.001	5.480	2.419–12.417	< 0.001
Pstage (≥T3)	2.642	2.034–3.432	< 0.001	2.202	1.592–3.044	< 0.001
PSM	1.793	1.417–2.270	< 0.001	1.601	1.193–2.148	0.002

BMI, Body mass index; PSA, prostate specific antige; Pstage, pathologic stage; PSM, positive surgical margin

### After propensity score matching.

After the matching, the significant relationship was consistently observed between the Ki-67 expression and several adverse pathologic outcomes [pathologic grade group (≥ 3) (HR 1.718, 95% CI 1.177–2.507, p = 0.005), pathologic stage (≥T3) (HR 1.707, 95% CI 1.162–2.507, p = 0.006), SVI (HR 1.826, 95% CI 1.041–3.202, p = 0.036), and PSM (HR 1.568, 95% CI 1.057–2.325, p = 0.025)(Table 2)]. The Kaplan-Meier analysis also revealed that the Ki-67 positive group had significantly inferior survival outcomes in terms of biochemical recurrence (p < 0.001, Fig 1) and subsequent multivariates Cox proportional analysis showed that the expression of Ki-67 was independently predicted the worse BCR-free survival in the propensity-score matched cohort as both categorical and continuous variables (Tables 3 and S1).

## Discussion

In this study, we could observe that the expression of Ki-67 was significantly associated with having postoperative adverse pathologic outcomes in the patients with localized prostate cancer. The expression of Ki-67 was also associated with worse BCR-free survival after radical prostatectomy in both entire and matched cohort in the Kaplan-Meier analysis and multivariates Cox-proportional analyses. We believe that our study is valuable in analyzing relatively large cohorts of localized prostate cancer solely treated by radical prostatectomy. Moreover we performed the propensity-score matching to elucidate the exact prognostic value of Ki-67 upon the other well-known strong biomarkers such as pathologic stage, PSA, and grade group.

Ki-67 protein was initially described by Gerdes et al in early 1980’s [11]. They demonstrated the novel protein and its initial prototype monoclonal antibody by using the tumor cells of Hodgkin’s lymphoma of mouse. Further characterization of Ki-67 antigen revealed that the antigen was exclusively reactive for the nuclear structure only in the proliferating cells, not in the quiescent or resting cells in the G_0_ phase of mitosis [12]. From this aspect, there were numerous attempts to utilize the Ki-67 antigen in diagnosing malignancies including breast, lung, brain, stomach, and pancreas cancers [13–18]. Nishimukai et al tried to evaluate the clinical impact of combination of progesterone receptor and Ki-67 expression in 327 patients with breast cancer [13]. They reported that the Ki-67 and progesterone receptor expression independently predicted the disease recurrence after surgery from their multivariate analyses. Another study by Inwald et al analyzed large database of 4,692 patients with breast cancer and found that Ki-67 expression was significantly related with higher tumor grading [14]. They also found significant relationship between high Ki-67 expression and worse prognosis (cancer-specific and overall survival) in patients with higher Ki-67 expression. Similar findings were observed in lung cancer. Warth et al analyzed the data of 1,065 patients with non-small cell lung cancer and revealed that the high Ki-67 index was evidently related with worse cancer-specific, and overall survival [15]. Moreover Khan et al and Zeng et al also demonstrated that the Ki-67 index can be utilized as a valuable prognostic biomarker in pancreas and brain tumor, subsequently [16–17].

Several groups previously endeavored to evaluate the prognostic value of Ki-67 index in the patients with prostate cancer [19–21]. Bettencourt analyzed the data of 180 localized prostate cancer patients treated by radical prostatectomy and reported that the high Ki-67 expression was significantly related with early progression and disease recurrence [19]. On the other hand, Pollack et al performed immunohistochemistry of Ki-67 using the pathologic specimen from 537 patients of Radiation Therapy Oncology Group database [20]. They found significant association between Ki-67 expression and worse survival outcomes (distant metastasis, and disease specific survival) in patients treated by radiotherapy. More recently, Fisher et al performed a retrospective review for 293 patients who were diagnosed by needle biopsy as localized prostate cancer and treated conservatively without any definitive treatment [21]. Biopsy grade group, PSA, age, extent disease and Ki-67 index were all significant predictor for cancer-specific mortality. Using the cutoff of 5% and 10% of Ki-67 index, they concluded that the Ki-67 index add significant additive clinical information in prediction of the prognosis of patients. Another study by Tretiakova et al analyzed data from 1000 prostatectomy specimens and found that the higher Ki-67 expression was strongly associated with higher grade group, stage, and high possibility of recurrence [22]. They also found that the high Ki-67 expression was also associated with higher rate of SVI and ECE. Furthermore, Malhotra et al analyzed the impact of three proliferation markers (Ki-67, DNA topoisomerase II, E2F transcription factor 1) and found that the each proliferation markers was revealed to be independently significant prognostic marker in their study [23]. When they combined the three biomarkers together as a “proliferation index”, it provided further superior prognostic performance with hazard ratio of 2.6. Another study by Fantony et al. analyzed the Ki-67 expression in 464 patients and found that there was significant difference in the Ki-67 expression level between the institutions [24]. They argued that standardization of staining process, interpretation and analyses is crucial to utilize the Ki-67 as a prognostic factor in PCa patients. Those aforementioned evidences support our results on the prognostic value of Ki-67 on predicting the patients’ disease outcomes. Moreover, we could observe the significant associations between high expression of Ki-67 and adverse pathologic outcomes from our multivariate analyses. As the high expression of Ki-67 implies the enhanced proliferation of the tumor cells, it would stand to reason that the high Ki-67 can represent the aggressive tumor with up-regulated growth. Lynn et al previously provided basic evidences showing that the expression of Ki-67 may be related with the abundant androgen and prolactin receptor, which can stimulate the production of Carboxypeptidase-D. The Carboxypeptidase-D is known to be involved in the intracellular NO production, which is known to have some roles in the progression, invasion, and angiogenesis of the tumor [25–27]. However, these findings should be validated by further studies.

Our study is limited by its retrospective design. Even though our data was accumulated by prospective fashion, there is some possibility for bias from retrospective data analysis. We should also admit that the possibility of selection bias may exist since our study only included the patients who treated by radical prostatectomy. Therefore, the clinical usefulness of Ki-67 in patients with metastatic disease or locally advanced disease should be evaluated by further study. Moreover, current study only evaluated the BCR-free survival without any mortality outcomes such as overall or cancer-specific survival due to the relatively short follow-up time, which is one of our major limitations. More importantly, the expression of Ki-67 was evaluated by one pathologist in this study, which is one of our main limitations. And the cut-off of Ki-67 in current study was comparably higher than the previous studies, which may due to different racial composition and/or inter-observer bias. But we strongly believe that the Ki-67 can provide some additive value for predicting the prognosis of localized prostate cancer.

## Conclusions

The expression of Ki-67 was significantly associated with worse pathologic and survival outcomes in patients with localized prostate cancer after treated by radical prostatectomy. These findings may help the future clinicians to predict the PCa prognosis more exactly and to choose the optimal treatments.

## Supporting information

S1 Table Multivariate analyses using Cox proportional hazard model on biochemical recurrence after propensity score matching (Ki-67 as continuous variable). (DOCX)

## Abbreviations:

RCC: renal cell carcinoma
BMI: body mass index
PCL: preoperative cholesterol level
OS: overall survival
CSS: cancer-specific survival
PFS: progression free survival
CT: computed tomography
ASA: American Society of Anesthesiologists
ROC: receiver operating curve
