# Supplementary material for: Elevated Ki-67 (MIB-1) expression as an independent predictor for unfavorable pathologic outcomes and biochemical recurrence after radical prostatectomy in patients with localized prostate cancer: A propensity score matched study
Source: PLoS One. 2019 Nov 7;14(11):e0224671. doi: 10.1371/journal.pone.0224671 (PMC6837325; doi:10.1371/journal.pone.0224671)
Supplement: S1 Table — (DOCX) [file pone.0224671.s001.docx]

| S1 Table 1. Multivariate analyses using Cox proportional hazard model on biochemical recurrence after propensity score matching (Ki-67 as continuous variable) | | | | | | |  |  |
| --- | --- | --- | --- | --- | --- | --- | --- | --- |
|  | Before propensity score matching | | | After propensity score matching | | |  |  |
|  | HR | 95% CI | p value | HR | 95% CI | p value |  |  |
| Age | 0.997 | 0.982 – 1.013 | 0.733 | 1.006 | 0.986 – 1.027 | 0.570 |  |  |
| BMI | 1.004 | 0.996 – 1.013 | 0.308 | 1.014 | 0.963 – 1.068 | 0.597 |  |  |
| Diabetes mellitus | 1.166 | 0.894 – 1.520 | 0.257 | 1.180 | 0.856 – 1.627 | 0.311 |  |  |
| Hypertension | 0.921 | 0.751 – 1.131 | 0.432 | 0.953 | 0.732 – 1.241 | 0.722 |  |  |
| PSA | 1.507 | 1.324 – 1.717 | < 0.001 | 1.608 | 1.351 – 1.914 | < 0.001 |  |  |
| Prostate volume | 0.994 | 0.986 – 1.001 | 0.107 | 0.996 | 0.989 – 0.998 | 0.048 |  |  |
| Ki-67 | 1.024 | 1.010 – 1.037 | 0.001 | 1.019 | 1.004 – 1.034 | 0.012 |  |  |
| Pathologic GS score |  |  |  |  |  |  |  |  |
| 6 | Reference | | | Reference | | |  | Reference |
| 7 | 2.914 | 1.604 – 5.295 | < 0.001 | 2.467 | 1.126 – 5.408 | 0.024 |  |  |
| ≥ 8 | 7.220 | 3.822 – 13.638 | < 0.001 | 5.571 | 2.456 – 12.638 | < 0.01 |  |  |
| Pathologic stage (≥pT3) | 2.686 | 2.068 – 3.488 | < 0.001 | 2.275 | 1.645 – 3.145 | < 0.001 |  |  |
| PSM | 1.820 | 1.439 – 2.303 | < 0.001 | 1.639 | 1.222 – 2.199 | 0.001 |  |  |
| BMI, Body mass index; PSA, prostate specific antige; GS, Gleason score; ECE, extracapsular extension; SVI, seminal vesicle invasion; PSM, positive surgical margin | | | | | | |  |  |
